# Resolution of dupilumab-associated alopecia areata with dosage modification

**DOI:** 10.1016/j.jdcr.2022.01.034

**Published:** 2022-02-17

**Authors:** Maansi Kulkarni, Craig A. Rohan, David Morris, Jeffrey B. Travers

**Affiliations:** a Department of Pharmacology and Toxicology, Boonshoft School of Medicine, Wright State University, Dayton, Ohio; b Department of Dermatology, Boonshoft School of Medicine, Wright State University, Dayton, Ohio; c Dayton VA Medical Center, Dayton, Ohio; d Department of Pediatrics, Boonshoft School of Medicine, Wright State University, Dayton, Ohio

**Keywords:** alopecia areata, atopic dermatitis, autoimmunity, dupilumab, AA, alopecia areata, AD, atopic dermatitis, BSA, body surface area, EASI, eczema area and severity index, IL, interleukin

## Introduction

Atopic dermatitis (AD) is one of the most common chronic skin conditions worldwide, affecting more than 15% of children and 2% of adults.^1^ Although AD has traditionally been treated with emollients and topical corticosteroids, newly developed immune-modifying drugs, such as dupilumab, an interleukin (IL) 4 and IL-13 receptor antagonist, have been added to the arsenal of treatment options.^2^

This case report describes a patient whose dupilumab use was associated with alopecia areata (AA). Although this phenomenon has been reported previously,^3^, ^4^, ^5^ our case report remains unique in that our patient demonstrated clinical improvement of his AD without hair loss when dupilumab was restarted at a modified dosage.^2^

## Case report

Our patient is a 22-year–old African American man with AD since infancy. In the past, the patient has been treated with topical corticosteroids and a Janus kinase inhibitor as part of a clinical trial as well as mycophenolate mofetil, with minimal improvement as measured by eczema area and severity index (EASI) scores. His past medical history includes an immunoglobulin E level >5000 IU/mL and a positive skin scratch test result to allergens from dog, cat, dust mites, mold, and peanut proteins. The patient has a positive family history of AD.

**Fig 1:**
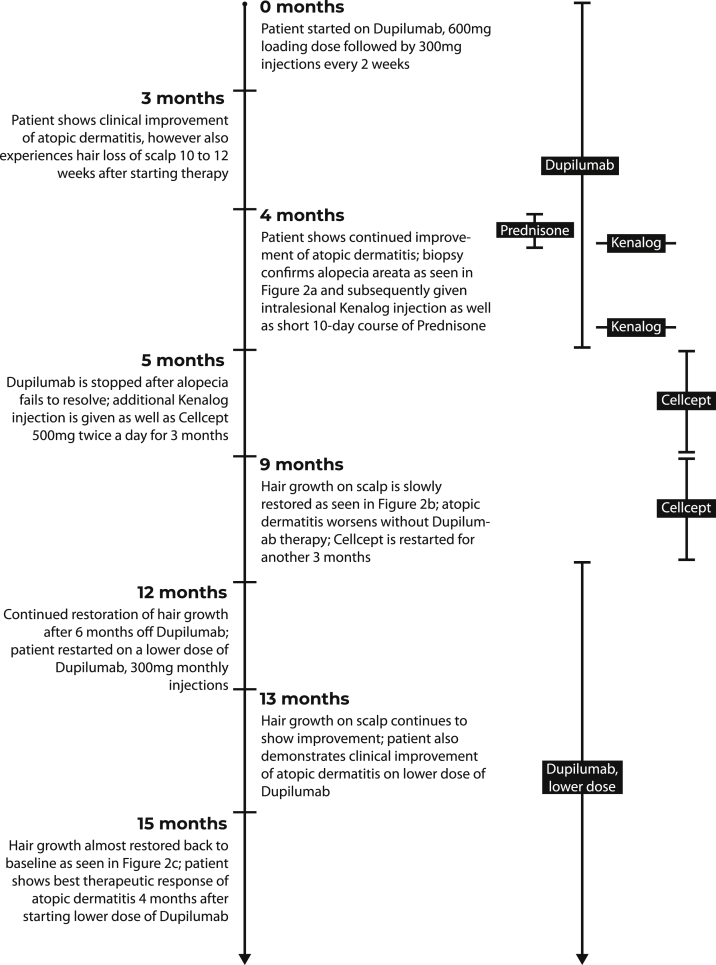
Detailed timeline of the patient’s clinical course.

With an EASI score of 30.6 and body surface area (BSA) involvement of 61%, the patient was considered to have moderate-to-severe AD, and thus dupilumab treatment was indicated. Per clinical guidelines, the patient was given a subcutaneous injection with a 600-mg loading dose, followed by 300-mg subcutaneous injections every 2 weeks. The patient’s detailed clinical course is summarized in Fig 1, and information on therapeutic responses to AD can be found in Table I.

**Table I:** Clinical course of atopic dermatitis in a patient treated with dupilumab

Highlights of the patient’s therapeutic clinical course	0 months	4 months	9 months	12 months	13 months	15 months
	Patient started on dupilumab, 600-mg loading dose followed by 300-mg injections every 2 weeks	Patient demonstrates clinical improvement of AD with development of alopecia areata; dupilumab is stopped at this time	6 months after stopping dupilumab, the patient’s hair growth returns, while AD worsens	The patient’s AD continues to worsen, and dupilumab is restarted, this time at a dose of 300 mg monthly	The new regimen shows improvement of AD, while the hair on the scalp continues to grow back	The patient demonstrates greatest therapeutic response to a lower dose of dupilumab, and the scalp hair is restored almost to baseline level
EASI	30.6	5.6	21.9	21.9	4.8	1.8
Head	2.8	0.6	2.1	0.21	0.6	0
Upper extremity	9.6	1.6	4.2	4.2	1.2	0.6
Trunk	6.0	1.8	7.2	7.2	0.6	0
Lower extremity	12.2	1.6	8.4	8.4	2.4	1.2
BSA involvement	61%	17%	35%	40%	9%	3%

*AD,* Atopic dermatitis; *BSA,* body surface area; *EASI,* eczema area and severity index.

**Fig 2:**
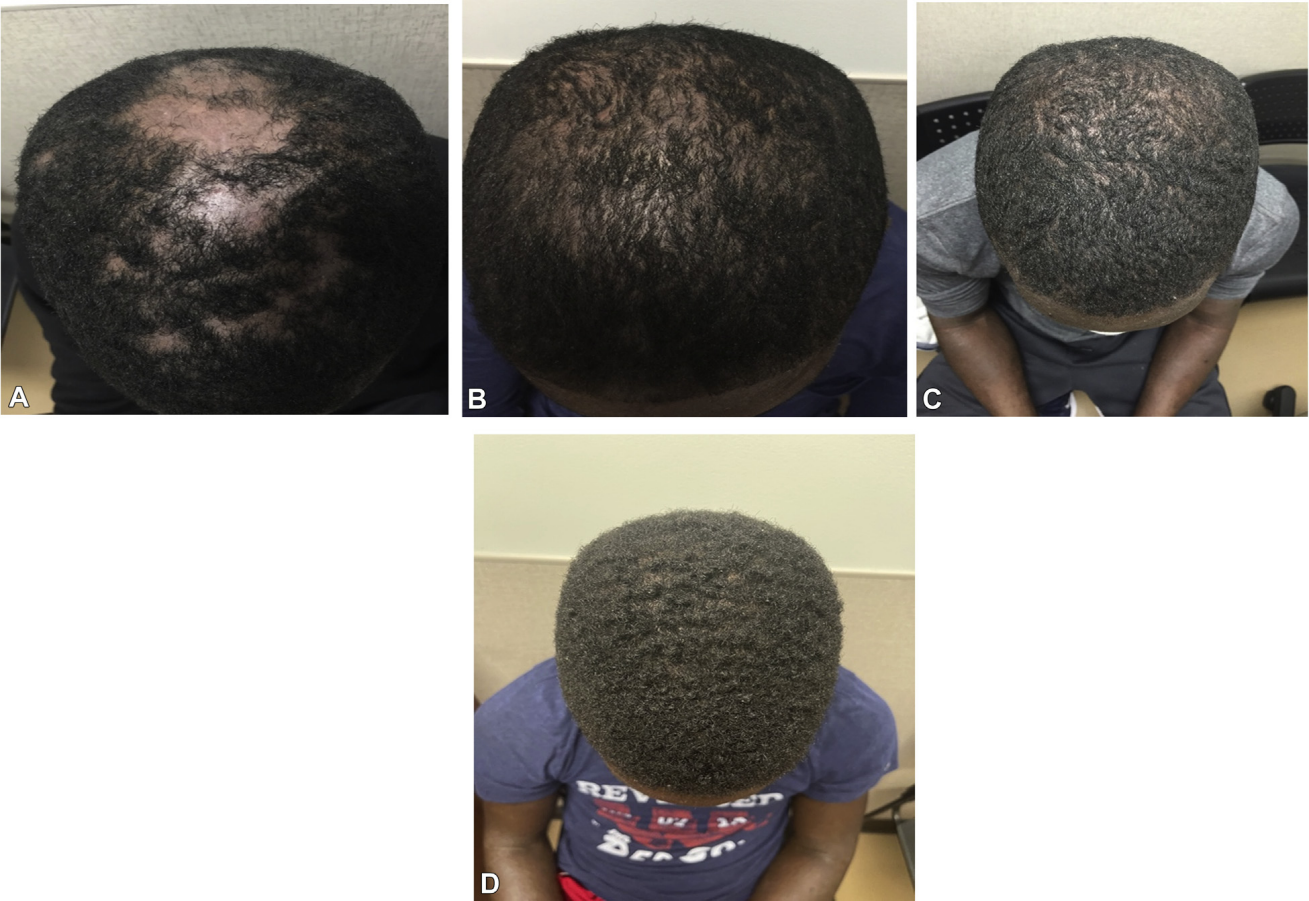
Clinical pictures documenting the patient’s scalp hair loss while on dupilumab. **A,** Hair loss of the scalp after 3 months on dupilumab (600-mg loading dose followed by 300-mg injections every 2 weeks). **B,** Hair growth 5 months after stopping dupilumab. **C,** Restoration and preservation of hair 4 months after starting a new dupilumab regimen, 300 mg monthly. **D,** Restoration of hair at 1 year on new low-dose dupilumab regimen.

The patient noted significant clinical improvement of his AD within 4 months of starting dupilumab (EASI, 5.6; BSA involvement, 17%). Unfortunately, the patient also experienced significant patchy hair loss localized to the vertex of the scalp (Fig 2, *A*). Biopsy results demonstrated spongiotic dermatitis with loss of hair follicles and perifollicular inflammation, consistent with AA. The patient was subsequently administered intralesional triamcinolone to the scalp, topical clobetasol ointment, as well as a short course (10 days) of oral prednisone. When hair growth was not noted even after a month, the patient was given another intralesional steroid injection to the area. Dupilumab was discontinued, and the patient was started on 500-mg mycophenolic acid mofetil twice daily for 3 months. Laboratory tests for other comorbid autoimmune conditions (antinuclear antibody, antithyroglobulin antibody, thyroid-stimulating hormone) were normal.

Within 6 months, the patient’s scalp hair slowly returned (Fig 2, *B*). During this time, the patient received approximately 1 more month of treatment with mycophenolate mofetil. Although slightly improved from baseline, the patient’s AD worsened. Six months after stopping dupilumab, his EASI score was 21.9 and BSA involvement was 35%.

Because of the clinical worsening of his AD, the patient requested to be restarted on dupilumab. Given the recalcitrant nature of his AD, after discussion of the potential side effects to include return of his AA or other potential autoimmune disorders, he was restarted on dupilumab, this time at a lower dose of 300 mg monthly. Four months after starting this new regimen, the patient showed significant improvement of his AD (EASI, 1.8; BSA involvement, 3%). Of note, the patient did not experience any additional hair loss with the new regimen, and his scalp hair returned to baseline (Fig 2, *C*). One year after restarting the lower dosage of dupilumab, he has had no recurrence of AA (Fig 2, *D*).

## Discussion

AD treatment has changed drastically because of the development of new immune-modifying drugs. Dupilumab has demonstrated an excellent therapeutic response in patients with refractory and severe AD.^6^ Dupilumab is a monoclonal antibody that interferes with IL-4 and IL-13 signaling, pathways thought to play a key role in the pathogenesis of AD.^7^ Dupilumab is a relatively new treatment option, being approved by the Food and Drug Administration for use in moderate-to-severe AD in 2017. Hence, data regarding dupilumab in a clinical context continue to evolve. For example, although more commonly reported adverse side effects—including injection site reactions, conjunctivitis, and reactivation of latent infections—are well known, very little is known about rarer adverse events associated with dupilumab use.^7^

This report documents a rare case of autoimmune hair loss with dupilumab use. Only a handful of case reports concerning autoimmune, endocrine, and dermatologic conditions with dupilumab use exist.^2^^,^^4^^,^^5^^,^^8^^,^^9^ Of note, Carnicle et al^5^ reported a similar scenario, in which dupilumab was used in the setting of AD and resulted in the reactivation of latent AA. However, in contrast to our case, this patient’s hair regrew with triamcinolone injections and continuation of dupilumab.^5^ Thus, the relationship between dupilumab use and autoimmunity appears nuanced and highly individualistic. To complicate this relationship, Gruenstein et al^4^ described a case in which dupilumab was used to concomitantly treat AD and AA successfully in a pediatric patient.

This case report also provides insights into clinical guidelines and dosing. Although our patient experienced a therapeutic response with the recommended dupilumab dosage of a 600-mg loading dose followed by 300-mg doses every 2 weeks, this regimen appears to be what triggered our patient’s AA. It subsequently took 6 months without dupilumab and aggressive therapy with mycophenolic acid mofetil and topical/intralesional corticosteroids to regain hair growth. Furthermore, our patient experienced his best therapeutic response with regard to his AD without hair loss when the maintenance dose was adjusted to 300 mg monthly. We propose that the ability of dupilumab to effectively block T helper 2 cell responses can lead to an increased incidence of autoimmunity by dysregulating T helper cell homeostasis. However, this relationship remains poorly understood, because there are reports suggesting that T helper 2 cell blockade has also shown to improve autoimmune conditions.^4^ Based on the complexity of this relationship between dupilumab and autoimmunity, not enough is known about dupilumab-associated autoimmune disorders to allow specific recommendations. Thus, further studies are needed to understand optimal dosing in the patients that experience adverse events to dupilumab.

## Conflicts of interest

None disclosed.
